# Efficacy and safety of acupuncture treatment as an adjunctive therapy after knee replacement: Single-center, pragmatic, randomized, assessor blinded, pilot study

**DOI:** 10.1097/MD.0000000000024941

**Published:** 2021-03-12

**Authors:** Tae-Yong Park, Hye-Jung Kim, Jin-Hyun Lee, Yun-Young Sunwoo, Kwang-Sun Do, Seong-Nim Han, Yun-Kyung Song, Dong-Sik Chae

**Affiliations:** a Institute for Integrative Medicine, Catholic Kwandong University International St. Mary's Hospital; b Department of Family, Catholic Kwandong University International St. Mary's Hospital, Catholic Kwandong University College of Medicine; c Iksoodang Korean Medical Clinic, Incheon; d SongHeon R&D, Apgujeong-ro, Sinsa-dong, Gangnam-gu, Seoul; e Department of Korean Rehabilitation Medicine, College of Korean Medicine, Gachon University, Seongnam-si; f Department of Orthopedic Surgery, Catholic University of Korea, Incheon St. Mary's Hospital, Incheon, South Korea.

**Keywords:** Acupuncture, adjunctive therapy, postoperative management, rehabilitation, total knee replacement

## Abstract

**Introduction:**

Total knee replacement (TKR) is a surgical procedure that is being increasingly performed as a result of population aging and the increased average human life expectancy in South Korea. Consistent with the growing number of TKR procedures, the number of patients seeking acupuncture for relief from adverse effects, effective pain management, and the enhancement of rehabilitative therapy effects and bodily function after TKR has also been increasing. Thus, an objective examination of the evidence regarding the safety and efficacy of acupuncture treatments is essential. The aim of this study is to verify the hypothesis that the concurrent use of acupuncture treatment and usual care after TKR is more effective, safe, and cost-effective for the relief of TKR symptoms than usual care therapy alone.

**Methods/design:**

This is an open-label, parallel, assessor-blinded randomized controlled trial that includes 50 patients with TKR. After screening the patients and receiving informed consent, the patients are divided into two groups (usual care + acupuncture group and usual care group); the patients will then undergo TKR surgery and will be hospitalized for 2 weeks. The patients will receive a total of 8 acupuncture treatments over 2 weeks after surgery and will be followed up at 3, 4, and 12 weeks after the end of the intervention. The primary outcome is assessed using the Korean version of the Western Ontario and McMaster Universities Arthritis Index (K-WOMAC), and the secondary outcome is measured using the Numerical Rating Scale (NRS), Risk of Fall, and Range of Motion (ROM). Moreover, the cost per quality-adjusted life years (QALYs) is adopted as a primary economic outcome for economic evaluation, and the cost per NRS is adopted as a secondary economic outcome.

**Ethics and dissemination:**

This trial has received complete ethical approval from the Ethics Committee of Catholic Kwandong University International St. Mary's Hospital (IS17ENSS0063). We intend to submit the results to a peer-reviewed journal and/or conferences.

**Trial registration:**

ClinicalTrials.gov Identifier: NCT03633097.

## 1 Introduction

Total knee replacement (TKR) is a surgical procedure that is being increasingly performed due to population aging and increased life expectancy in South Korea. The number of TKR procedures increased by over 135%, from 45,879 in 2015 to 62,344 in 2019. The medical cost of public health insurance associated with TKRs totaled 37,077,059,000 won (∼32,638,256 US dollars) in 2015 and increased to 55,499,427,000 won (∼48,850,829 US dollars) in 2019.^[1]^ The age distribution of patients undergoing TKR shows that 36.2% of the patients were aged 60 to 69 years, 48.6% were aged 70 to 79 years, and 8.7% were aged ≥80 years,^[2]^ indicating that TKR is primarily performed on elderly patients. A large number of patients undergo TKR due to degenerative knee arthritis. Consistent with the high incidence of TKR in South Korea, the number of patients seeking acupuncture for pain management and enhancement for rehabilitative therapy effects and bodily function not only before but also after TKR has also been increasing.

In the western-medicine-based clinical settings in South Korea, acupuncture treatment after TKR is considered unsafe regardless of the acupuncture points in use because of the risk of infection and reoperation, and the practice is often rejected or prohibited. Furthermore, according to a recent systematic review, including acupuncture in a rehabilitation program is beneficial as an adjunct to opioids for pain management across several types of postsurgical conditions.^[3]^ However, the results of studies investigating the effect of acupuncture after TKR are not in concordance.^[4–7]^ Moreover, no previous study has examined the efficacy, safety, and cost-effectiveness of post-TKR acupuncture rather than focusing solely on its efficacy.

Therefore, it is worth assessing the effectiveness, safety, and cost-effectiveness of acupuncture as an adjunctive therapy in patients following TKR in a pilot study.

## 2 Objective

The aim of this study is to verify the hypothesis that the concurrent use of acupuncture treatment and usual care after TKR is more effective, safe, and cost-effective relieving TKR symptoms than usual care therapy alone.

## 3 Methods

### 3.1 Trial registration

This study has been registered at ClinicalTrials.gov (trial registration number: NCT03633097; trial protocol version 2.0; https://clinicaltrials.gov/ct2/show/NCT03633097).

### 3.2 Study design

**Figure 1:**
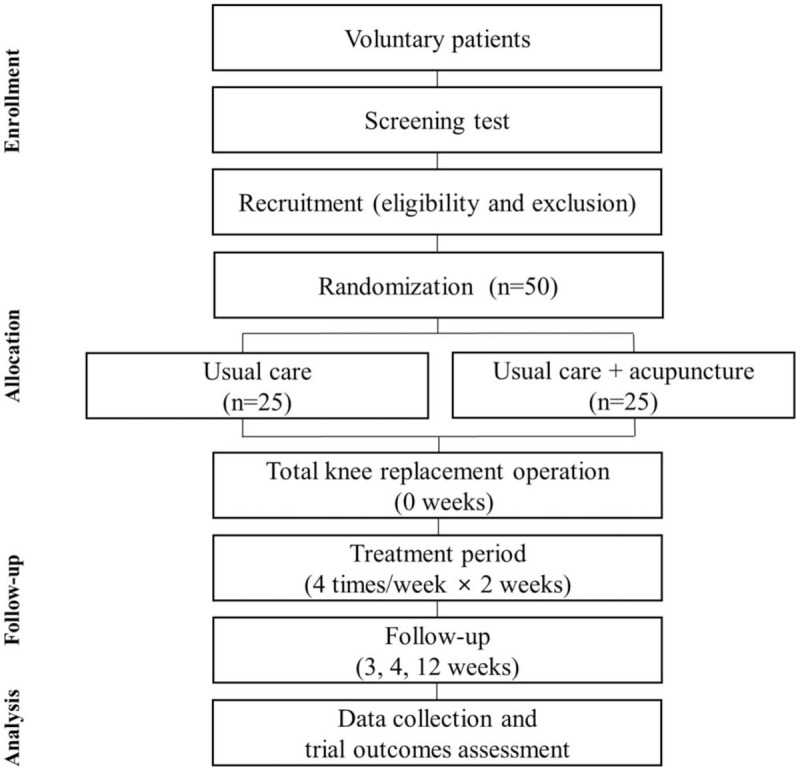
Study Flow Chart.

This study was designed as an open-label, parallel, assessor-blinded randomized controlled trial. This study will be conducted at Catholic Kwandong University International St. Mary's Hospital in Incheon, South Korea. The clinical trial and the items to be examined are presented in Figure 1 and Table 1. The patients will receive a full explanation of the trial details from investigators. Through this procedure, if they agree to participate in the trial, a signed consent form will be obtained.

**Table 1:** Clinical trial schedule and observation items.

	Study period											Follow-up		
	Screening	W1					W2					W3	W4	W12
Tasks	V1	OP	V2	V3	V4	V5	V6	V7	V8	V9	V10^∗^	F/U1^†^	F/U2	F/U3
Obtaining consent forms from participants	●													
Demographic investigation	●													
History investigation (medical history, previous medication use)	●													
Participant screening	●													
Randomization	●													
Acupuncture + UC			●	●	●	●	●	●	●	●	●^‡^			
UC			●	●	●	●	●	●	●	●	●^‡^			
Efficacy test														
K-WOMAC	●										●		●	
NRS	●		●				●				●	●	●	●
Risk of fall	●										●			●
ROM	●						●				●		●	●
Safety assessment														
Vital signs	●		●	●	●	●	●	●	●	●	●		●	●
Adverse reaction assessment			●	●	●	●	●	●	●	●	●	●	●	●
Laboratory test	●					●				●			●	●
Investigation on the doses of medications that can be administered if needed													●	●
Treatment compliance assessment											●			
Investigation on concomitant medications	●		●	●	●	●	●	●	●	●	●	●	●	●
Investigation on blindness maintenance (examiner)											●			
Cost-effectiveness evaluation	●										●		●	●

<visit schedule: window period included>	Visit 7: 6 days (POD 9 ± 2 day)
Visit 1: 7 days to −1 day from Visit 2	Visit 8: 7 days (POD 10 ± 2 days)
Visit 2: 0 days (POD 3 ± 2 days)	Visit 9: 8 days (POD 11 ± 2 days)
Visit 3: 1 day (POD 4 ± 2 days)	Visit 10: 3 days within Visit 9
Visit 4: 2 days (POD 5 ± 2 days)	FU 1: 3 weeks within Visit 2 (21 days ± 5 days)
Visit 5: 3 days (POD 6 ± 2 days)	FU 2: 4 weeks within Visit 2 (28 days ± 5 days)
Visit 6: 5 days (POD 8 ± 2 days)	FU 3: 12 weeks within Visit 2 (84 days ± 5 days)

OP = operation, POD = postoperative day, UC = usual care.

∗ Previous visit ± 3 days.

† FU1 will be performed through a phone call.

‡ PRN medications needed during the FU period are provided to both groups on the day of discharge.

The patients visited the hospital five times for evaluation. The screening (Visit 1, V1) included only those participants who submitted the informed consent form. For screening, the demographic information, medical history, physical examination, vital signs, questionnaire survey, laboratory test, and selection/exclusion criteria will be evaluated. Randomization and group allocation will be performed at screening. Fifty patients were enrolled and randomly divided into two groups. Patients who have passed the screening test will have a TKR and will be hospitalized in ∼2 weeks with usual care or usual care plus acupuncture therapy.

The patients will receive a total of eight acupuncture treatments over 2 weeks after surgery (four sessions between 3 and 8 days after surgery and four sessions between 9 and 14 days after surgery). They will be followed up at 3, 4, and 12 weeks after the end of the intervention. The primary outcomes will be assessed at V1, V10, and Week 4 of follow-up. The safety evaluations include adverse reaction evaluations, vital sign assessments, and laboratory tests. The adverse reaction evaluation will be performed every day during the hospitalization period and continued during follow-up at 3, 4, and 12 weeks after surgery. The vital sign assessment will also be performed daily during the hospitalization period after surgery and at 4 and 14 weeks of the follow-up. Laboratory tests will be performed during screening, at V5 and V9, and at 4 and 12 weeks of follow-up. An economic evaluation will be performed during the treatment period (2 weeks) and at 4 and 12 weeks after surgery and will investigate medical costs and utility (quality of life) to estimate the quality-adjusted life years (QALYs). The Euro-Qol five dimension scale (EQ-5D), a multiattribute health measurement instrument, will be used to assess the quality of life at baseline (Week 1) and at 2 and 4 weeks. Medical costs will also be investigated at baseline (week 1) and at 2, 4, and 12 weeks for a total of four assessments. These evaluations are performed simultaneously with the clinical trial through in-person interviews during the survey period and through in-person interviews, phone calls, and online communication using questionnaires thereafter according to a predefined arrangement with the patient.

The coordinators of the trial will inform the patient of the next visit schedule for each visit and encourage participation. The clinical trial conductor will instruct the participant to ensure compliance with the treatment.

Only those patients who completed the informed consent form with full acceptance of the expected effects and predicted adverse reactions to clinical trial interventions prior to the commencement of the study may participate in the study. In the event of an accident or adverse event, appropriate measures will be taken according to the investigator's judgment, and the participant will be compensated. Moreover, the study will be conducted ethically in compliance with the principles of the Good Clinical Practice (GCP) guidelines and the revised version of the Declaration of Helsinki.

### 3.3 Sample size calculation

In this clinical trial, changes in the K-WOMAC will be analyzed and assessed to examine the effect of acupuncture on pain and functional improvements in patients who underwent TKR.

Due to the lack of information about domestic clinical trials to examine the K-WOMAC as a primary outcome for assessing the effect of acupuncture on the knee joint, we referred to a study in which aromatherapy massage was performed on patients with knee arthritis (married women).^[8]^

The changes in the K-WOMAC after 2 weeks of aromatherapy massage or regular massage in the experimental and control groups were −6.10 ± 4.05 and 0.24 ± 4.16, respectively. The difference in the K-WOMAC between the groups was 6.34. In this study, the difference in the K-WOMAC between the experimental and control groups is assumed to be 3.60. We referred to previous studies for standard deviations. The level of statistical significance is set at 0.05 (α, two-tailed), and power (1−β) is set at 80%.

The minimum sample size needed to test the statistical significance was calculated using ‘G∗Power 3.1.9.2’ and was found to be 22 participants per group. Considering a 10% dropout rate, 25 participants per group or a total of 50 participants will be recruited.

### 3.4 Intervention

#### 3.4.1 Usual care group

The critical pathway (CP) for TKR (Supplemental Digital Content [Appendix 1]) used at the orthopedics department of our institution will be followed as a standard. However, depending on the patient's condition, adjustments may be made for pharmacotherapy and rehabilitative therapy. Rehabilitative physical therapy and pharmacotherapy will be performed according to the schedule proposed in the CP.

##### 3.4.1.1 Rehabilitative therapy following TKR (physiotherapy)

Three days after surgery, the patients will start rehabilitation with continuous passive motion and knee extension, quadriceps femoris strengthening exercises, and gait rehabilitation.

1. Continuous passive motion (CPM) of the knee

Patients place their operated leg on a knee joint exercise machine and perform rehabilitative exercises for 30 min after entering their range of flexion in a digital machine. The range of flexion is determined based on the range of motion over which pain at the surgical site is tolerable.

2. Quadriceps femoris-strengthening exercises

Quadriceps set: Patients use their thigh muscles to extend their knee joints for 5 to 10 min and rest for several seconds. This set will be repeated ∼10 times. At least 10 sets are recommended per day.

Straight leg raises: Patients bend the unoperated leg in the prone position and extend the operated leg. They are required to raise the operated leg 15 cm from the floor and maintain their position for 5 to 10 s. This exercise will be repeated 10 times, and at least 10 sets are recommended per day.

Ankle pumps: The muscles in the lower limbs are contracted to pull the feet toward the head, and the position is maintained for 5 to 10 s. After each exercise, the patients rest for several seconds. This exercise will be repeated 10 times. At least 10 sets are recommended per day.

3. Gait rehabilitation

Patients begin walking carefully using a walker or crutches at 3 days after surgery and gradually put more weight on their legs. The body weight is evenly distributed on the walker or crutches while the patients are standing in a comfortable position. The walker and crutches are moved forward by a short distance, and the operated leg is completely extended to allow the heel of the foot to touch the floor first as the patients walk forward. It is recommended that they walk for short periods of time several times during the day since more than 30 min of continuous walking can aggravate swelling or pain.

##### 3.4.1.2 Pharmacotherapy

Pharmacotherapy will be performed in accordance with the TKR CP of the orthopedics department of Catholic Kwandong University International St. Mary's Hospital (Supplemental Digital Content [Appendix 1]).

##### 3.4.1.3 Concomitant drugs and combination therapy/contraindicated medications

Patients will be required to confirm with the principal investigator whether they can take specific medications or receive treatments concomitantly with acupuncture before taking or receiving them. The name, dosage, dose, and duration of use of any concomitant drugs that are administered will be recorded on the case report form.

Physiotherapy can be performed concomitantly with acupuncture at the discretion of the participant even during the follow-up period.

#### 3.4.2 Acupuncture treatment group

The Acu-treatment group is treated with acupuncture, in addition to the treatment for the usual care therapy group. A disposable sterilized needle made of stainless steel (0.2 mm × 30 mm, Spring Handle Needle, DONGBANG Medical Co, LTD) will be used for treatment. The acupuncture points for treatment are GB 29, GB 28, BL 38, ST 36, CV 3, SP 9, GB 34, ST 28, SP 10, and ST 34. All the acupuncture points were used on the TKR-operated side for 20 min. The reporting of this randomized study was based on the Standards for Reporting Interventions in Controlled Trials of Acupuncture (STRICTA). The Acu-treatment group will receive eight treatments (four sessions between 3 and 8 days after surgery and four sessions between 9 and 14 days after surgery) over 2 weeks. A licensed doctor of Oriental medicine with formal education in the field and over 5 years of clinical experience and acupuncture will perform acupuncture according to the intervention schedule proposed in the protocol.

### 3.5 Inclusion and exclusion criteria

#### 3.5.1 Inclusion criteria

The inclusion criteria are as follows:

1. adult men and women aged 60 years and older and
2. patients with a diagnosis of osteoarthritis and listed for unilateral TKR;
3. those with normal cognitive function; and
4. patients who are willing and able to give informed consent for participation in the study.

#### 3.5.2 Exclusion criteria

The following patients are excluded from participating in the trial:

1. those who had undergone total knee arthroplasty due to gout;
2. those who had total knee arthroplasty due to trauma;
3. those who had total knee arthroplasty due to autoimmune-related diseases such as rheumatoid arthritis;
4. those who were participating in other clinical trials;
5. those who had a history of participating in other clinical trials within 1 month;
6. those who had difficulty writing a research consent form; and
7. those who were judged to be inappropriate for the clinical study by the researchers.

### 3.6 Recruitment, randomization, blinding, and nonblinding

Advertisements for patient recruitment will be posted on the Catholic Kwandong University International St. Mary's Hospital bulletin boards, along with posters, banner advertisements and internet cafes. All advertisements will be run after IRB approval.

Individuals who have given written consent for participating in the study will be assigned a screening number in order of the outpatient visit at screening. Unblinded subinvestigators who do not influence the research results and analysis will refer to the randomization list that has been prepared by a commissioned professional statistician and will assign participation numbers. Participants will be allocated to the usual care group and the usual care plus acupuncture group at a ratio of 1:1. A block randomization table is generated by an independent statistician, and patients are assigned to one of two groups according to the randomization table. The screening numbers, randomization numbers, and initials assigned to each participant are used as subject identification codes to identify the participant until the end of the clinical trial.

The usual care used in this study is not a clinical investigation product and does not require allocation concealment, since it is administered to all the groups. Moreover, it is impossible to blind the investigator and the participant in each group due to the nature of the study intervention, which is based on the contact between the patient and the medical staff. Instead, the investigator maintains blinding to prevent bias in evaluating the participant. If the clinical trial is completed, all clinical report forms (CRFs) are collected, the database process is terminated, and unblinding will be performed if required for statistical analysis.

### 3.7 Rescue therapy and concomitant medications

If a participant experiences unbearable pain from screening to discharge, he/she may be allowed to take any rescue medication according to the judgment of the medical staff. The rescue medication was as follows in the hospital or after discharge:

1. pethidine HCl 25 mg,
2. oxycodone hydrochloride 5 mg, and
3. tramadol hydrochloride.

If the participant takes any rescue medication, the date and dose of administration will be recorded in the study participant's log. If concomitant medication is administered during the trial period, the name of the drug, the daily dose, and the duration of administration should be noted on the CRFs.

### 3.8 Study outcomes

#### 3.8.1 Primary outcome

The primary outcome in this study was the changes from the baseline in the K-WOMAC. The time frame was as follows: screening, day 11 (visit 10, V10), and day 21 (follow-up 2, FU2). The K-WOMAC^[9]^ was used to evaluate the physical function. The WOMAC is an index that evaluates the pain and functional status of patients with degenerative knee arthritis and is widely used as a measure for evaluating symptoms and treatment effects in patients with degenerative knee joints. It is a self-administered questionnaire consisting of 24 items divided into 3 subscales.^[9]^

#### 3.8.2 Secondary outcome

The secondary outcome assessment measures the change from the baseline in the numerical rating scale (NRS). The time frame is as follows: screening, baseline, and days 5, 11, 21, 28, and 84. The NRS range is 0 to 10, with 0 being no pain and 10 being the worst pain possible. In addition, the following outcome assessments are surveyed as a secondary outcome measurement: Change from the baseline in a physical condition related to mobility, which is a 5-m walking test at a comfortable speed of −5 mWT (meters/second) at every visit (time frame: screening, days 11 and 84); change from the baseline in a physical condition related to endurance, which is a sit-to-stand test (seconds) at every visit (time frame: screening, days 11, 84); change from the baseline in the timed up and go test, which is a functional test for evaluating the time to rise from a chair, walk 3 m, turn around, walk back to a chair and sit down (abnormal cutoff value >12 s) at every visit (time frame: screening, day 11, day 84); change from the baseline in the alternative step test, which is a functional test measuring the clinical balance performance, and it has been shown to predict fall risk among elderly subjects (seconds) at every visit (time frame: screening, day 11, day 84); and change from the baseline in the range of motion test, which compares the flexion and extension angle using a long-arm goniometer between the experimental and comparator groups (time frame: screening, baseline, days 5, 11, 28, and 84).

#### 3.8.3 Safety and adverse events monitoring

The safety assessment includes

1. adverse reactions,
2. vital signs, and
3. clinical laboratory testing.

The subinvestigator will record the details of any adverse reactions and the use of concomitant medications during the clinical trial in the case report form. In case of an adverse reaction, the symptoms and signs of the adverse reaction, duration (start date/end date), severity, result, significance, causal relationship to the drug under clinical investigation, and measures taken for its alleviation will be recorded. The names, dosages, duration of administration, reason for administration, etc, of the concomitant medications will be recorded in detail.

Clinical laboratory tests will be performed at screening, V5, V9, FU2, and FU3. Since this study will evaluate the safety of the usual care and acupuncture combination, it differs from clinical studies that evaluate the safety and pharmacokinetic properties of drugs. Therefore, this study conducts clinical laboratory tests for the following items for the purpose of the standard safety lab test:

1. hematology: hemoglobin, hematocrit, red blood cell count, white blood cell count, and differential count, platelet count;
2. serum chemistry: glucose fasting, aspartate aminotransferase (AST), alanine aminotransferase (ALT), blood urea nitrogen (BUN), electrolytes (sodium, potassium, and chloride), serum creatinine (SCr), erythrocyte sedimentation rate (ESR), C-reactive protein (CRP); and
3. coagulation: D-dimer, fibrin degradation products (FDP);
4. γ-glutamyl transpeptidase (γ-GTP), alkaline phosphatase, creatinine phosphokinase (CPK), uric acid, total bilirubin, total protein, albumin, osteocalcin, carboxy-terminal collagen crosslinks (CTX), urine analysis: pH, protein (albumin), glucose, leukocyte esterase, and blood were performed only at screening.

#### 3.8.4 Early termination or dropout

The early termination or dropout criteria are as follows:

1. Violation of the inclusion/exclusion criteria
2. Withdrawal of consent for participation
3. Adverse reactions (including serious adverse reactions)

Adverse reactions refer to undesirable and unintended signs, symptoms, or diseases and do not necessarily have a causal relationship with the interventions administered during a clinical trial. However, since the present clinical trial involves patients who underwent TKR, adverse reactions may be deemed symptoms or side effects caused by TKR at the discretion of the operating surgeon and principal investigator. Symptoms that the principal investigator deems as unassociated with the intervention of this clinical trial are not classified as adverse reactions that occur during this clinical trial.

4. Poor compliance (compliance below 70% in the final compliance assessment) or inability to comply
5. Violation of the clinical trial protocol
6. Other reasons deemed valid by the researchers
7. Diagnosis of an infection in the recovery room

#### 3.8.5 Data collection, access, and management

All information regarding the patient is anonymized through initial processing, and all investigators are obliged to maintain the confidentiality of the results. The source document is registered immediately when the data are collected, and it will be recorded in the CRF. All trial documents will be kept safe, and only those who have been approved by the principal investigator will have access to all the data related to the trial.

The data management for this clinical trial will be conducted in accordance with the standard working guidelines of the Catholic Kwandong University Clinical Research Center, and other matters not specified in the protocol will be conducted in accordance with the International Council for Harmonisation of Technical Requirements for Pharmaceuticals for Human Use (ICH) guidelines for GCP and Korea-GCP standards.

### 3.9 Statistical analysis

#### 3.9.1 General principle for statistical analysis

An intention-to-treat (ITT) analysis will be performed as the primary analysis for patients who receive acupuncture at least once. A per-protocol analysis will also be performed for participants who have completed the study after excluding dropouts.

For intergroup comparisons, an independent *t* test will be performed for continuous variables if normality is satisfied. If normality is not satisfied, the Wilcoxon rank-sum test, a nonparametric statistical technique, will be performed. For comparisons between different time points within each group, a paired *t* test or Wilcoxon signed rank test will be used. An analysis of covariance (ANCOVA) adjusted for covariant factors that show significant differences with respect to the baseline in each treatment group and the initial baseline outcomes will be additionally determined. For categorical variables, the chi-square test or Fisher's exact test will be used.

All statistical analyses will be two-tailed with a 5% significance level. The last observation carried forward (LOCF) method will be used to handle missing values. Multiple imputation or a regression analysis will be additionally used to examine differences in the results.

#### 3.9.2 Demographic data

Continuous variables will be expressed as the means ± standard deviation (SD), and categorical variables will be expressed as n (%). The chi-square test and independent *t* test will be used to examine differences in basic demographic variables, including sex, age, occupation, and medical history.

#### 3.9.3 Outcome analysis

##### 3.9.3.1 Primary outcomes

The difference between the K-WOMAC score measured during screening and that measured after the last treatment session (V10) will be determined for each participant. The statistical significance of the difference in the K-WOMAC for each group will be determined using a paired *t* test or Wilcoxon signed rank test with a 5% statistical significance level. The independent *t* test or Wilcoxon rank sum test will be used to determine the statistical significance of the efficacy of acupuncture in each group. The results will be adjusted for covariant factors, which are factors that can affect the results of an analysis.

##### 3.9.3.2 Secondary outcomes

The paired *t* test will be used to analyze the changes in the NRS, risk of falling, and range of motion (ROM) from the baseline to V10 in each group. Student's *t* test will be used to analyze these changes between different groups. A repeated-measured ANOVA will be used to analyze the differences in the changes in all variables, including the K-WOMAC score that occur within a group before the treatment.

##### 3.9.3.3 Safety variables

The number of adverse reactions (vital signs, abnormal values in a physical test, acute reactions observed after vaccination, and local or systemic reactions) will be determined for each group, and their incidence and 95% confidence intervals will be presented. The chi-square test will be used for intergroup comparisons of adverse reactions.

Continuous data such as blood or blood chemistry test parameters, physical test parameters, and vital signs will be presented for each group and assessed using descriptive statistics. A paired *t* test will be used for comparisons before and after the treatment, and Student's *t* test will be used for intergroup comparisons. The frequency and percentage will be presented for categorical data such as urine test results, and McNemar's test will be used to determine normal and abnormal conditions for each visit.

##### 3.9.3.4 Economic evaluation parameters

###### 3.9.3.4.1 Target subjects

Patients who participated in the clinical trial at least once starting with V2 will be included in the economic evaluation.

###### 3.9.3.4.2 Economic evaluation parameters and criteria

The medical costs generated as a result of this clinical trial will be calculated through a medical cost analysis in the economic evaluation. If necessary, the health insurance and medical costs at the present institution may be used to represent a portion of the unit costs. A sensitivity analysis will be performed on the medical costs.

1. Primary economic outcomes
  (a) Cost per QALY gained
  (b) The EQ-5D will be used to estimate the quality of life needed to calculate the QALY. Quality of life will be used as the primary economic outcome. The area-under-the-curve method will be used to calculate the QALYs.
2. Secondary economic outcome
  (a) Effectiveness indices such as the cost per NRS
3. Economic evaluation period

The initial analysis will take 4 weeks. If further estimation is necessary after 4 weeks, the costs and effectiveness will be extrapolated using a regression model, or secondary analyses such as a decision analysis will be performed.

Medical costs generated as a result of this clinical trial will be calculated based on the number of treatment sessions and unit costs. Researchers will determine and record the medical costs generated inside the clinical trial institution by reviewing the electronic records after they are unblinded.

If the time horizon exceeds 12 months, the unit of cost will be unified to the South Korean won of 2017, and a 5% discount will be applied in accordance with the economic evaluation guideline by the Health Insurance Review and Assessment Service. The analytical point of view of this study is society's point of view. The representative values (e.g., mean, etc) of the parameters used in this study will be used in a baseline analysis. A probabilistic sensitivity analysis will be performed using the distributions of all the estimated parameters and their representative values.

###### 3.9.3.4.3 Handling the data from dropouts

For drop-outs, the last obtained measurements will be used for all the remaining visits to make it seem as if the measurements were obtained during those visits (LOCF: last-observation-carried-forward analysis).

###### 3.9.3.4.4 Exceptions to the exclusion criteria for the economic evaluation

- Participants who drop out or are excluded due to the treatment effects or adverse effects of acupuncture or due to the worsening of the disease will be subject to an economic evaluation as long as the evaluation is feasible. The economic evaluation will be continued for the following dropout cases due to treatment effects or adverse effects.
- Cases: Drop-out due to recovery or loss of symptoms during the treatment period, drop-out due to the adverse effects from a drug, and treatment-related drop-out deemed by our researchers as associated with the target disease or the treatment provided in this clinical trial due to the secondary occurrence of a disease (e.g., surgical site infections, etc).
- These exceptional cases will be included in the ITT analysis as there is a high possibility that the effect estimates will go missing during the study. The LOCF will again be used to estimate the missing values. However, in cases in which the missing pattern is not monotone, multiple imputation can be used to check the sensitivity.

### 3.10 Quality control and data monitoring

This study will be monitored by SongHeon R&D Co, Ltd, a Contract Research Organization (CRO) company that consults with institutions conducting clinical trials to ensure compliance with the protocol and K-GCP. During monitoring, crosschecks will be made with the evidence to ensure that the documents (trial master files, CRF, informed consent forms, and adverse event reports) are complete and clear.

### 3.11 Ethics and dissemination

#### 3.11.1 Research ethics approval

This trial has received complete ethical approval from the Ethics Committee of Catholic Kwandong University International St. Mary's Hospital (IS18ENSI0063).

#### 3.11.2 Protocol amendments

The investigators who want protocol amendments should first discuss it with the principal investigator, and they can change the protocol after obtaining approval from the IRB. However, when a dangerous situation occurs and immediate care is needed, the protocol change will be reported to the IRB at a later time.

#### 3.11.3 Posttrial care

If the patients experience unexpected accidents or injuries, they will receive appropriate medical care at Catholic Kwandong University International, St. Mary's Hospital. Additionally, appropriate compensation will be made by the insurance company, according to the patient compensation rules of the trial.

## 4 Discussion

TKR is known to be effective for moderate to severe knee arthritis,^[10]^ but it does not completely ameliorate the symptoms or discomfort in all patients. A majority of the patients who receive TKR are elderly individuals with weakness or reduced strength in the muscles around the knees due to prolonged knee arthritis, and they are unable to manage their pain or restore their functions due to the inability to receive the rehabilitative therapy required after TKR. Furthermore, due to the nature of TKR, elderly patients typically receive TKR after a long-period of knee arthritis. For this reason, they may suffer from systemic weakness and delayed recovery after TKR, and they often have poor pain management and functional recovery leading to a poor quality of life.

In South Korea, patients often receive a complementary alternative treatment (Oriental medicine) after TKR to reduce postoperative discomfort. Previous studies attempted to approach pain or stiffness management after TKR using an adjunctive complementary alternative treatment. Case reports have been published reporting two cases of herbal medicine therapy for pain and stiffness after TKR^[11]^ and one case of degenerative knee arthritis treated with TKR that was relieved by herbal medicine therapy.^[12]^ Aside from the case reports, however, no systematic randomized controlled trials (RCTs) that use evidence-based medical methodologies have been conducted. To overcome this limitation, we plan to analyze the efficacy, safety, and cost-effectiveness of acupuncture comprehensively for post-TKR management. We believe that this study will be instrumental in providing better alternative solutions for TKR management.

## Author contributions

Tae-Yong Park and Hye-Jung Kim contributed to the trial design and the writing of the manuscript. Jin-Hyun Lee, Yun-Young Sunwoo, and Kwang-Sun Do provided perspectives on the selection of the trial interventions and advice on the trial procedure. Seong-Nim Han contributed to a monitoring and a document management. Both Dong-Sik Chae and Yun-Kyung Song are equally responsible for writing the manuscript and managing and supervising the clinical trial. All the authors have read and approved the final version of the manuscript.

**Conceptualization:** Yun-Kyung Song, Yun-Young Sunwoo, Dong-Sik Chae.

**Data curation:** Seong-Nim Han.

**Formal analysis:** Yun-Young Sunwoo.

**Funding acquisition:** Yun-Kyung Song.

**Investigation:** Dong-Sik Chae.

**Methodology:** Yun-Kyung Song, Jin-Hyun Lee, Kwang-Sun Do.

**Project administration:** Yun-Kyung Song, Dong-Sik Chae.

**Resources:** Seong-Nim Han.

**Software:** Kwang-Sun Do, Seong-Nim Han.

**Supervision:** Dong-Sik Chae.

**Visualization:** Kwang-Sun Do.

**Writing – review & editing:** Yun-Kyung Song, Hye-Jung Kim.

**Writing – original draft:** Tae-Yong Park, Hye-Jung Kim.

## Supplementary Material

Supplemental Digital Content

## References

[1] Service. HIRA. Healthcare big data hub; 2020. Available at: http://opendata.hira.or.kr/op/opc/olapDiagBhvInfo.do [accessed October 21, 2020].

[2] Guideline Center for Korean Medicine. Korean Medicine Clinical Practice Guideline for Post-operative treatment of total knee arthroplasty. [Internet] 2017. Available at: http://www.nckm.or.kr/nckm/module/practiceGuide/viewPDF.do?guide_idx=100.

[3] Wu MS Chen KH Chen IF. The efficacy of acupuncture in post-operative pain management: a systematic review and meta-analysis. PLoS One 2016;11:e0150367. 26959661 10.1371/journal.pone.0150367 PMC4784927

[4] Chen CC Yang CC Hu CC. Acupuncture for pain relief after total knee arthroplasty: a randomized controlled trial. Reg Anesth Pain Med 2015;40:31–6. 25158837 10.1097/AAP.0000000000000138

[5] Crespin DJ Griffin KH Johnson JR. Acupuncture provides short-term pain relief for patients in a total joint replacement program. Pain Med 2015;16:1195–203. 25586769 10.1111/pme.12685 PMC4478153

[6] Mikashima Y Takagi T Tomatsu T. Efficacy of acupuncture during post-acute phase of rehabilitation after total knee arthroplasty. J Tradit Chin Med 2012;32:545–8. 23427386 10.1016/s0254-6272(13)60068-0

[7] Tsang RC Tsang PL Ko CY. Effects of acupuncture and sham acupuncture in addition to physiotherapy in patients undergoing bilateral total knee arthroplasty—a randomized controlled trial. Clin Rehabil 2007;21:719–28. 17846072 10.1177/0269215507077362

[8] Inja Kim E-KK. Effects of aroma massage on pain, activities of daily living and fatigue in patients with knee osteoarthritis. J Muscle Joint Health 2009;16:145–53.

[9] Tae-Sung Ko S-YK Jong-Soo Lee. Reliability and validity of the Korean Western Ontario and McMaster Universities (WOMAC) osteoarthritis index in patients with osteoarthritis of the knee. J Oriental Rehab Med 2009;19:251–5.

[10] Skou ST Roos EM Laursen MB. A randomized, controlled trial of total knee replacement. N Engl J Med 2015;373:1597–606. 26488691 10.1056/NEJMoa1505467

[11] Yo-Han Kim MHH Jae-Soo Kim Hyun-Jong Lee. Two cases report of Korean Medical Treatment for pain and stiffness after total knee replacement. J Oriental Spine Joint 2016;13:91–8.

[12] Kim C-G Jo D-C Moon S-J. Korean Medical Rehabilitation for Total Knee Replacement. J Korean Med Rehabil 2014;24:111–8.

