# Supplementary material for: Efficacy and safety of acupuncture treatment as an adjunctive therapy after knee replacement: Single-center, pragmatic, randomized, assessor blinded, pilot study
Source: Medicine (Baltimore). 2021 Mar 12;100(10):e24941. doi: 10.1097/MD.0000000000024941 (PMC7969218; doi:10.1097/MD.0000000000024941)
Supplement: Supplemental Digital Content [file medi-100-e24941-s001.docx]

Appendix 1. TKR inpatients Critical Pathway

|  | The other day of OP | OP date  (Day of surgery) | | POD #1 | POD #2 | POD #3 | POD #4 | POD #5 |
| --- | --- | --- | --- | --- | --- | --- | --- | --- |
|  |  | Pre-OP | Post-OP |  |  |  |  |  |
| Activity | No limit | No limit | ABR | Wheel chair ambulation | Start to walk using walker | Walking exercise using walker | Walking exercise using walker | Walking exercise using walker |
| Management &  Monitoring |  |  |  |  |  | CPM | CPM | CPM |
| Lab |  |  | CBC 6 unit  Electro 4 unit  AST/ALT, BUN/Cr, Glucose | CBC 6 unit  Electro 4 unit  AST/ALT, BUN/Cr, Glucose,  Albumin | CBC 6 unit  Electro 4 unit  AST/ALT, BUN/Cr, Glucose, ESR, CRP, D-dimer, FDP, Bacterial culture | CBC 6 unit  Electro 4 unit  AST/ALT, BUN/Cr, Glucose, ESR, CRP, D-dimer, FDP, Albumin |  |  |
| X-ray |  |  | Knee AP & Lat  Knee both Obl |  |  |  |  | Bone density  Diagnostic fluoroscopy  Long bone both |
| Fluid |  |  | NS10-C 1PK#1 IV (80 cc/hr) | NS10-C 1PK#1 IV (40 cc/hr)  Amino combination 1PK#1 IV (40 cc/hr) | NS10-C 1PK#1 IV (40 cc/hr) | NS10-C 1PK#1 IV (40 cc/hr) | NS10-C 1PK#1 IV (40 cc/hr) |  |
| Antibiotics |  |  | Cefbuperazone dihydrate 1 g | Cefbuperazone dihydrate 1 g  2 V#2 (12 hrs, 24 hrs after OP) |  |  |  |  |
| Medication | Celecoxib 200 mg 1C#1 PO |  | Metoclopramide hydrochloride hydrate 2A #2 + NS1-C 2PK #2  Celecoxib 200 mg 2C # 2PC PO  Salbutamol sulfate 2.5 1 ea+ sodium chloride 2 ml IHM  <PRN>  propacetamol hydrochloride 1 V # 1+NS1-C 1PK #1 | Ramosetron hydrochloride 0.1 ㎎ 1A#1 IV  Esomeprazole strontium tetrahydrate 20 mg 1T#1PC  Acetaminophen 162.5 ㎎+ Tramadol hydrochloride 18.75 ㎎ 3T#3PC  Celecoxib 200 ㎎ 1C#1PC | Esomeprazole strontium tetrahydrate 20 mg 1T #1  Acetaminophen 162.5 ㎎+Tramadol hydrochloride 18.75 ㎎ 3T#3  Celecoxib 200 ㎎ 1C#1 | Enoxparin sodium 40 mg  Esomeprazole strontium tetrahydrate 20 mg 1T #1  Acetaminophen 162.5 ㎎+Tramadol hydrochloride 18.75 ㎎ 3T#3  Oxycodone hydrochloride 10 mg 2T # 2PC PO | Enoxparin sodium 40 mg  Esomeprazole strontium tetrahydrate 20 mg 1T #1  Acetaminophen 162.5 ㎎+Tramadol hydrochloride 18.75 ㎎ 3T#3  Oxycodone hydrochloride 10 mg 2T # 2PC PO | Enoxparin sodium 40 mg  Esomeprazole strontium tetrahydrate 20 mg 1T #1  Acetaminophen 162.5 ㎎+Tramadol hydrochloride 18.75 ㎎ 3T#3  Oxycodone hydrochloride 10 mg 2T # 2PC PO |
| Pain Control |  |  | Tramadol hydrochloride 100 mg 3A # 3 IV+NS1-C 3PK #3 IV  Oxycodone hydrochloride 2T # 2PC PO  <PRN>  Pethidine HCl 25 mg IM | Tramadol hydrochloride 100 mg 3A # 3 IV+NS1-C 3PK #3 IV  <PRN>  Oxycodone hydrochloride 5 mg 2T # 2PC PO  Pethidine HCl 25 mg IM | Tramadol hydrochloride 100 mg 3A # 3 IV+NS1-C 3PK #3 IV  <PRN>  Oxycodone hydrochloride 5 mg 2T # 2PC PO  Pethidine HCl 25 mg IM | Tramadol hydrochloride 100 mg 3A # 3 IV+NS1-C 3PK #3 IV  <PRN>  Pethidine HCl 25 mg IM | <PRN>  Tramadol hydrochloride 100 mg 1A # 1 IV+NS1-C 1PK #1 IV  Pethidine HCl 25 mg IM | <PRN>  Tramadol hydrochloride 100 mg 1A # 1 IV+NS1-C 1PK #1 IV  Pethidine HCl 25 mg IM |
| Education  Consultation |  |  |  |  |  |  | Q-set  ROM exercise | Q-set  ROM exercise |

|  | POD #6 | POD #7 | POD #8 | POD #9 | POD #10 | POD #11 | POD #12  (HD 14days)  Discharge |
| --- | --- | --- | --- | --- | --- | --- | --- |
| Activity | Walking exercise using walker | Walking exercise using walker | Walking exercise using walker | Walking exercise using walker | Walking exercise using walker | Walking exercise using walker | Walking exercise using walker |
| Management &  Monitoring | CPM | CPM | CPM  Elastic stocking supply | CPM  Elastic stocking supply | CPM  Elastic stocking supply | CPM  Elastic stocking supply | Elastic stocking supply |
| Lab | CBC 6 unit  Electro 4 unit  AST/ALT, BUN/Cr, Glucose, ESR, CRP, D-dimer, FDP, Albumin  PT, PTT* |  |  |  |  | CBC 6 unit  Electro 4 unit  AST/ALT, BUN/Cr, Glucose, ESR, CRP, D-dimer, FDP, Albumin  PT, PTT* | <OPD FU>  CBC 6 unit  Electro 4 unit  AST/ALT, BUN/Cr, Glucose, ESR, CRP, D-dimer, FDP, Albumin, intact PTH, 25-(OH) Vitamin D, CTx, Osteocalcin, Cr, Co |
| X-ray |  |  |  |  |  |  | <OPD FU>  Knee AP & Lat X-ray |
| Antibiotics |  |  |  |  |  |  |  |
| Medication | Enoxparin sodium 40 mg  Esomeprazole strontium tetrahydrate 20 mg 1T #1  Acetaminophen 162.5 ㎎+Tramadol hydrochloride 18.75 ㎎ 3T#3  Oxycodone hydrochloride 10 mg 2T # 2PC PO  Vit D 5 ㎎/㎖ 1A IM | Enoxparin sodium 40 mg  Esomeprazole strontium tetrahydrate 20 mg 1T #1  Acetaminophen 162.5 ㎎+Tramadol hydrochloride 18.75 ㎎ 3T#3  Oxycodone hydrochloride 10 mg 2T # 2PC PO | Enoxparin sodium 40 mg  Esomeprazole strontium tetrahydrate 20 mg 1T #1  Acetaminophen 162.5 ㎎+Tramadol hydrochloride 18.75 ㎎ 3T#3  Oxycodone hydrochloride 10 mg 2T # 2PC PO | Enoxparin sodium 40 mg  Esomeprazole strontium tetrahydrate 20 mg 1T #1  Acetaminophen 162.5 ㎎+Tramadol hydrochloride 18.75 ㎎ 3T#3  Oxycodone hydrochloride 10 mg 2T # 2PC PO | Enoxparin sodium 40 mg  Esomeprazole strontium tetrahydrate 20 mg 1T #1  Acetaminophen 162.5 ㎎+Tramadol hydrochloride 18.75 ㎎ 3T#3  Oxycodone hydrochloride 10 mg 2T # 2PC PO | Enoxparin sodium 40 mg  Esomeprazole strontium tetrahydrate 20 mg 1T #1  Acetaminophen 162.5 ㎎+Tramadol hydrochloride 18.75 ㎎ 3T#3  Oxycodone hydrochloride 10 mg 2T # 2PC PO | Meloxicam 7.5 ㎎ 2T #2 for 80 days  Duloxetine hydrochloride 30 mg 1C#1 for 80 days  Esomeprazole strontium tetrahydrate 20 mg 1T #1  For 80days  micronized rivaroxaban 10㎎ 1T#1 for 14days  Oxycodone hydrochloride 5 mg PRN PO for 20days |
| Pain Control | <PRN>  Tramadol hydrochloride 100 mg 1A # 1 IV+NS1-C 1PK #1 IV  Pethidine HCl 25 mg IM | <PRN>  Tramadol hydrochloride 100 mg 1A # 1 IV+NS1-C 1PK #1 IV  Pethidine HCl 25 mg IM | <PRN>  Tramadol hydrochloride 100 mg 1A # 1 IV+NS1-C 1PK #1 IV  Pethidine HCl 25 mg IM | <PRN>  Tramadol hydrochloride 100 mg 1A # 1 IV+NS1-C 1PK #1 IV  Pethidine HCl 25 mg IM | <PRN>  Tramadol hydrochloride 100 mg 1A # 1 IV+NS1-C 1PK #1 IV  Pethidine HCl 25 mg IM | <PRN>  Tramadol hydrochloride 100 mg 1A # 1 IV+NS1-C 1PK #1 IV  Pethidine HCl 25 mg IM | <PRN>  Tramadol hydrochloride 100 mg 1A # 1 IV+NS1-C 1PK #1 IV  Pethidine HCl 25 mg IM |
| Education  Consultation | Q-set  ROM exercise | Q-set  ROM exercise | Q-set  ROM exercise | Q-set  ROM exercise | Q-set  ROM exercise | Q-set  ROM exercise |  |

OP: Operation, POD: Postoperative Day, ABR: Absolutely Bed Rest, CPM: Continuous Passive Motion,

| AP: Anteroposterior, Lat: Lateral, Obl: Oblique, EA: each,  *: The patients who will be prescribed enoxaparin must provide their PT, PTT test, CBC: complete blood count, Electro: Electrolyte, AST: Aspartate Aminotransferase, ALT: Alanine Aminotransferase, BUN: Blood Urea Nitrogen, ESR: Erythrocyte Sedimentation Rate, CRP: C-Reactive Protein, FDP: Fibrin Degradation Product, T: Tablet, C: Capsule, A: Ampule, OPD: Out-Patient Department, PO: By Mouth, Orally, ROM: Range of Motion, PRN: As Required (Lat. pro re nata), IV: Intra-Venous injection, IM: Intra-Muscular injection, and PK: Pack | | |  |
| --- | --- | --- | --- |
|  | |  |  |
|  |  |  |  |
